# Real-time correction of channel-bed roughness and water level in river network hydrodynamic modeling for accurate forecasting

**DOI:** 10.1038/s41598-023-42791-x

**Published:** 2023-11-24

**Authors:** Yifan Chen, Feifeng Cao, Weiping Cheng, Bin Liu, Pubing Yu

**Affiliations:** 1 Zhejiang Institute of Hydraulics & Estuary (Zhejiang Institute of Marine Planning and Design), Hangzhou, 310017 China; 2 Zhejiang Provincial Key Laboratory of Hydraulic Disaster Prevention and Mitigation, Hangzhou, 310017 China; 3 College of Civil Engineering, Zhejiang University of Technology, Hangzhou, 310023 China; 4 College of Civil Engineering and Architecture, Zhejiang University, Hangzhou, 310058 China; 5 College of Traffic and Transportation Engineering, Changsha University of Science and Technology, Changsha, 410114 China

**Keywords:** Natural hazards, Civil engineering

## Abstract

The accuracy and reliability of hydrodynamic models are sensitive to both hydraulic state variables and model parameters, particularly the bed roughness, while their simultaneous real-time corrections and corresponding effects still need to be well-established and understood. This paper presents a real-time data assimilation model that corrects channel-bed roughness and water level in a river network hydrodynamic model, ensuring its accuracy and reliability. Experiments and parameter analysis evaluated the effect of initial roughness and observation noise level on model performance. Correcting both roughness and water level improved filtering time and forecasting accuracy by up to 63% and 80%, respectively, compared to methods only correcting water level. The filtering time was reduced by 44–63%, and the water level forecasting RMSE decreased by up to 80%. Both models experienced increased filtering time and forecasting error as observation noise increased, but the proposed model had a lower increase. With accurate hydraulic state measurement (e.g., 0.005 m error), the model achieved negligible water level forecasting error after 7 h of data assimilation. The model's accuracy depended on the initial channel-bed roughness, and the algorithm enables real-time roughness correction, making it useful for flood forecasting.

## Introduction

River networks are crucial in practice as they play a critical role in many aspects of society and the environment^1, 2^. They provide crucial ecosystem services such as water supply for drinking, irrigation, and industrial purposes, support for transportation, recreation and tourism, and habitat for various plant and animal species. In recent years, there has been a surge of efforts in the simulation and prediction of river network flow by developing river network hydrodynamic models coupling with a series of data assimilation methods or intelligent methods such as Kalman filter (or extended Kalman filter) and genetic algorithm^3–6^. The Kalman filter was developed by Kálmán in the late 1950s and early 1960s^7^. It is a recursive filter that estimates the state of a linear dynamic system from a series of noisy measurements. The Kalman filter is optimal under the assumptions that the system dynamics and measurement models are linear, the noise is Gaussian and white, and the initial state and noise covariance matrices are known. The Extended Kalman Filter (EKF) was later developed as an extension of the Kalman filter to handle nonlinear systems^8^. The EKF linearizes the system dynamics and measurement models about the current state estimate and then applies the standard Kalman filter update equations. This approach works well for "almost linear" systems but can give poor results for highly nonlinear systems.

Compared to single open-channel rivers, the water flow simulation in river networks is more complex, particularly for turbulent water flow in mountain river networks, as it is impacted by many factors such as the shape and slope of the river channel and surrounding land surface, river bed roughness, channel geometry, hydraulic states (e.g., discharge, water level), water temperature, meteorological conditions (e.g., precipitation, evaporation), and human activities. In some cases, the need for more local flow data as prior data could also limit the water flow simulation capacity.

Many research efforts have been devoted to improving the performance of river network hydrodynamic models based on Saint–Venant equations and Navier–Stokes equations, the models of which can be roughly divided into the unit division model, node-channel model, and hybrid model^9, 10^. To date, researchers have found that applying hydrodynamic models with constant model parameters or hydraulic state variables in the flow simulation process fails to produce an acceptable flood forecasting accuracy for a relatively long period (e.g., for more than 24 h). The data assimilation treatment and the real-time correction of model parameters and hydraulic state variables are thus developed^11–13^.

The real-time calibration of river network hydrodynamic models encompasses estimating state variables and model parameters. A method was established to accurately monitor daily evapotranspiration (ET) by combining a coupled energy-water budget model and a data assimilation scheme. This indicated that a dual state-parameter filter provided optimal results^14^. Further, various methods have been proposed to account for other parameters, such as the non-uniformity of velocity distribution in the momentum equation^15, 16^. Among the hydrodynamic model parameters, the bed roughness has attracted many research interests. For example, the bed roughness factor caused by vegetation in natural rivers was estimated using data assimilation. The results indicate that the magnitude of the roughness coefficients in each subregion was aligned with the vegetation species in the area, reflecting the flow resistance associated with the vegetation^17^. Similarly, Forzieri et al.^18^ calibrated a 1D numerical hydraulic model using Quickbird images and LIDAR data, showing the effect of different vegetation types and patterns on the hydraulic roughness parameter. In addition, a remote sensing technique was used to calibrate hydrodynamics models. The roughness coefficient was estimated without the need for image ortho-rectification. It found that the optimum roughness coefficient depends on discharge, decreasing as the flow rate increases^19^.

The roughness parameter plays a significant role in the hydrodynamic model. It is an important hydraulic parameter that reflects the comprehensive influence of factors such as roughness of flow perimeter, flow plane shape, section geometry, vegetation condition and hydraulic condition change on flow resistance. That is, it strictly has a spatiotemporal variation rather than being fixed. Most simulations are based on the Reynolds-averaged Navier–Stokes equations, where roughness effects have been accounted for by roughness functions determined empirically from experiments^20^. To improve the model accuracy and save computational cost, researchers proposed the virtual boundary method^21–23^. In the virtual boundary method, the rough wall, composed of individual, geometrically predefined roughness elements, is embedded in a Cartesian grid, and the no-slip condition on the immersed elements is imposed by appropriate body forces. In these three studies, the shape and distribution of the roughness elements were known a priori and were resolved by several grid points. Uncertainty exists in the cross-sectional areas of rivers during flood periods, with changes to the geometry being especially common after flood events. Hydrological processes in an estuary are particularly nonlinear and intricate^24^. As a result, an appropriate value for the roughness coefficient could significantly improve the accuracy of a hydraulic model.

This paper aims to establish a real-time hydrodynamic correction model of the river network with the simultaneous real-time correction of channel-bed roughness and water level by using the extended Kalman filtering technique. The performance of a water level forecasting model is evaluated experimentally with a parameter analysis to determine the impact of initial roughness and observation noise on its accuracy and filtering time. The results of this study are compared to those of a data assimilation model that only employs real-time correction of the state variable, i.e., water level. The understanding gained will help in the real-time optimization of hydrodynamic process simulation and prediction for complex river networks.

## River network hydrodynamic system and data assimilation

Saint Venant equations are often used to describe the one-dimensional flow, as follows:

Continuous equation:

1 $$\frac{\partial A}{\partial t}+\frac{\partial Q}{\partial x}={q}_{L}$$

Momentum equation:

2 $$\frac{{\partial Q}}{{\partial t}} + \frac{\partial }{{\partial x}}\left( {\frac{{\alpha Q^{2} }}{A}} \right) + gA\frac{{\partial Z}}{{\partial x}} + gA\frac{{Q\left| Q \right|}}{{K^{2} }} = q_{L} v_{x}$$

where *A is cross-*section *area*; *t* is time; *Q* is average flow of cross-section; *x* is displacement along the water flow; *q*_*L*_ is lateral streamflow per unit length; *a* is momentum correction coefficient;* g* is the acceleration of gravity;* Z* is the water level of section; *K* is the flow modulus, *K* = *AR*^*2/3*^*/n*, where* R* is the hydraulic radius of the river and *n* is the Manning coefficient; *v*_*x*_ is the velocity component of the lateral stream flow along the water flow, usually taken as *v*_*x*_ = 0.

Saint Venant equations are hyperbolic partial differential equation for which analytical solutions are not yet available. The finite difference method is an effective numerical discretization method for solving the Saint Venant equations and is divided into two schemes: the explicit difference scheme and the implicit difference scheme. Although the explicit difference scheme does not require a joint solution of equations, the time step and space step are limited by strict adherence to the "Courant condition" in order to maintain stability, whereas the implicit difference scheme is more flexible in the selection of the time and space step and is therefore popularly used. In this paper, the Preissmanm four-point implicit difference scheme is adopted to discretize the equations, and the space–time discretization scheme of the continuous and momentum equations are obtained as follows:

Difference form for the continuous equations:

3 $$\begin{aligned} & \frac{{\theta \left( {\Delta B_{{i + 1}} + \Delta B_{i} } \right) + \left( {B_{i}^{t} + B_{{i + 1}}^{t} } \right)}}{2} \cdot \frac{{\Delta Z_{{i + 1}} + \Delta Z_{i} }}{{2\Delta t}} + \left( {\theta \frac{{\Delta Q_{{i + 1}} - \Delta Q_{i} }}{{\Delta x}} + \frac{{Q_{{i + 1}}^{t} - Q_{i}^{t} }}{{\Delta x}}} \right) \\ & \quad = \frac{{\theta \left( {\Delta q_{{Li}} + \Delta q_{{Li + 1}} } \right) + \left( {q_{{Li}}^{t} + q_{{Li + 1}}^{t} } \right)}}{2} \\ \end{aligned}$$

Difference form for the momentum equations:

4 $$\begin{aligned} & \frac{{\Delta Q_{i + 1} + \Delta Q_{i} }}{2\Delta t} + \frac{\theta }{\Delta x}\left[ {\frac{{(Q_{i + 1}^{t} + \Delta Q_{i + 1} )^{2} }}{{A_{i + 1}^{t} + \Delta A_{i + 1} }} - \frac{{(Q_{i}^{t} + \Delta Q_{i} )^{2} }}{{A_{i}^{t} + \Delta A_{i} }}\left] { + \frac{1 - \theta }{{\Delta x}}} \right[\frac{{(Q_{i + 1}^{t} )^{2} }}{{A_{i + 1}^{t} }} - \frac{{(Q_{i}^{t} )^{2} }}{{A_{i}^{t} }}} \right] \\ & \quad + \frac{{\Delta Q_{i + 1} + \Delta Q_{i} }}{2\Delta t} + \frac{\theta }{\Delta x}\left[ {\frac{{(Q_{i + 1}^{t} + \Delta Q_{i + 1} )^{2} }}{{A_{i + 1}^{t} + \Delta A_{i + 1} }} - \frac{{(Q_{i}^{t} + \Delta Q_{i} )^{2} }}{{A_{i}^{t} + \Delta A_{i} }}\left] { + \frac{1 - \theta }{{\Delta x}}} \right[\frac{{(Q_{i + 1}^{t} )^{2} }}{{A_{i + 1}^{t} }} - \frac{{(Q_{i}^{t} )^{2} }}{{A_{i}^{t} }}} \right] \\ & \quad + \left[ {\frac{g\theta }{2}\left( {\Delta A_{i + 1} + \Delta A_{i} } \right) + \frac{g}{2}\left( {A_{i + 1}^{t} + A_{i}^{t} } \right)} \right]\left[ {\frac{\theta }{\Delta x}\left( {\Delta Z_{i + 1} - \Delta Z_{i} } \right) + \frac{1}{\Delta x}\left( {Z_{i + 1}^{t} - Z_{i}^{t} } \right)} \right] \\ & \quad + \frac{g\theta }{2}\left[ {\frac{{\left( {A_{i + 1}^{t} + \Delta A_{i + 1} } \right)\left( {Q_{i + 1}^{t} + \Delta Q_{i + 1} } \right)\left| {Q_{i + 1}^{t} + \Delta Q_{i + 1} } \right|}}{{(K_{i + 1}^{n} + \Delta K_{i + 1} )^{2} }} + \frac{{\left( {A_{i}^{t} + \Delta A_{i} } \right)\left( {Q_{i}^{t} + \Delta Q_{i} } \right)\left| {Q_{i}^{t} + \Delta Q_{i} } \right|}}{{(K_{i}^{n} + \Delta K_{i} )^{2} }}} \right] \\ & \quad + \frac{{g\left( {1 - \theta } \right)}}{2}\left[ {\frac{{A_{i + 1}^{t} Q_{i + 1}^{t} \left| {Q_{i + 1}^{t} } \right|}}{{(K_{i + 1}^{n} )^{2} }} + \frac{{A_{i}^{t} Q_{i}^{t} \left| {Q_{i}^{t} } \right|}}{{(K_{i}^{n} )^{2} }}} \right] = 0 \\ \end{aligned}$$

where *θ* is the Preissmanm weighting factor; superscripts *t* and* t* + 1 and subscripts i and *i* + *1* represent the time and one-dimensional spatial layers respectively; *B* is the river width. Thus, the linear equations expressed in increments is obtained:

5 $$\left\{\begin{array}{c}{a}_{1i}{\Delta Q}_{i}+{b}_{1i}\Delta {Z}_{i}+{c}_{1i}\Delta {Q}_{i+1}+{d}_{1i}\Delta {Z}_{i+1}={e}_{1i}\\ {a}_{2i}{\Delta Q}_{i}+{b}_{2i}\Delta {Z}_{i}+{c}_{2i}\Delta {Q}_{i+1}+{d}_{2i}\Delta {Z}_{i+1}={e}_{2i}\end{array}\right.$$

where

6 $$\begin{aligned} a_{1i} = & - 4\frac{\theta \Delta t}{{\Delta x_{i} }}\frac{1}{{B_{i}^{t} + B_{i + 1}^{t} }};\;b_{1i} = 1;\;c_{1i} = 4\frac{\theta \Delta t}{{\Delta x_{i} }}\frac{1}{{B_{i}^{t} + B_{i + 1}^{t} }};\;d_{1i} = 1; \\ e_{1i} = & 2\frac{{\Delta t\left( {q_{Li}^{t} + q_{Li + 1}^{t} } \right)}}{{B_{i + 1}^{t} + B_{i}^{t} }} - 4\frac{\Delta t}{{\Delta x_{i} }}\frac{{Q_{i + 1}^{t} - Q_{i}^{t} }}{{B_{i + 1}^{t} + B_{i}^{t} }};\;a_{2i} = 1 - 4\frac{\theta \Delta t}{{\Delta x_{i} }}\frac{{Q_{i}^{t} }}{{A_{i}^{t} }} + 2\theta \Delta tg\frac{{A_{i}^{t} \left| {Q_{i}^{t} } \right|}}{{(K_{i}^{t} )^{2} }}; \\ b_{2i} = & \frac{\theta \Delta t}{{\Delta x_{i} }}\left[ {2\frac{{\left( {Q_{i}^{t} } \right)^{2} B_{i}^{t} }}{{\left( {A_{i}^{t} } \right)^{2} }} - g\left( {A_{i}^{t} + A_{i + 1}^{t} } \right) + g\left( {Z_{i + 1}^{t} - Z_{i}^{t} } \right)B_{i}^{t} } \right] + g\theta \Delta t\frac{{Q_{i}^{t} \left| {Q_{i}^{t} } \right|}}{{(K_{i}^{t} )^{2} }}\left( {B_{i}^{t} - 2\frac{{A_{i}^{t} }}{{K_{i}^{t} }}\frac{{dK_{i}^{t} }}{{dZ_{i}^{t} }}} \right); \\ c_{2i} = & 1 + 4\frac{\theta \Delta t}{{\Delta x_{i} }}\frac{{Q_{i + 1}^{t} }}{{A_{i + 1}^{t} }} + 2\theta \Delta tg\frac{{A_{i + 1}^{t} \left| {Q_{i + 1}^{t} } \right|}}{{(K_{i + 1}^{t} )^{2} }}; \\ d_{2i} = & \frac{\theta \Delta t}{{\Delta x_{i} }}\left[ { - 2\frac{{\left( {Q_{i + 1}^{t} } \right)^{2} B_{i + 1}^{t} }}{{\left( {A_{i + 1}^{n} } \right)^{2} }} + g\left( {A_{i}^{t} + A_{i + 1}^{t} } \right) + g\left( {Z_{i + 1}^{t} - Z_{i}^{t} } \right)B_{i + 1}^{t} } \right] + g\theta {\Delta }t\frac{{Q_{i + 1}^{t} \left| {Q_{i + 1}^{t} } \right|}}{{(K_{i + 1}^{t} )^{2} }}\left( {B_{i + 1}^{t} - 2\frac{{A_{i + 1}^{t} }}{{K_{i + 1}^{t} }}\frac{{dK_{i + 1}^{t} }}{{dZ_{i + 1}^{t} }}} \right); \\ e_{2i} = & \frac{\Delta t}{{\Delta x_{i} }}\left[ { - 2\frac{{\left( {Q_{i + 1}^{t} } \right)^{2} }}{{A_{i + 1}^{t} }} + 2\frac{{\left( {Q_{i}^{t} } \right)^{2} }}{{A_{i}^{t} }} - g\left( {A_{i + 1}^{t} + A_{i}^{t} } \right)\left( {Z_{i + 1}^{t} - Z_{i}^{t} } \right)} \right] - g\Delta t\left[ {\frac{{A_{i + 1}^{t} Q_{i + 1}^{t} \left| {Q_{i + !}^{t} } \right|}}{{(K_{i + 1}^{t} )^{2} }} + \frac{{A_{i}^{t} Q_{i}^{t} \left| {Q_{i}^{t} } \right|}}{{(K_{i}^{t} )^{2} }}} \right]; \\ \end{aligned}$$

*A*_*1i*_ ~ *E*_*2i*_ are the expressions for each of the known quantities in time layer *t*. After elimination of the elements, we can obtain:

7 $$\left\{\begin{array}{c}\Delta {Z}_{i+1}={a}_{1i}{\prime}\Delta {Z}_{i}+{b}_{1i}{\prime}\Delta {Q}_{i}+{c}_{1i}{\prime}\\ \Delta {Q}_{i+1}={a}_{2i}{\prime}\Delta {Z}_{i}+{b}_{2i}{\prime}\Delta {Q}_{i}+{c}_{2i}{\prime}\end{array}\right.$$

where

8 $$\begin{aligned} a_{1i}{\prime} = & \frac{{c_{2i} b_{1i} - c_{1i} b_{2i} }}{{c_{1i} d_{2i} - c_{2i} d_{1i} }};\; b_{1i}{\prime} = \frac{{c_{2i} a_{1i} - }}{{c_{1i} d_{2i} - c_{2i} d_{1i} }};\; c_{1i}{\prime} = \frac{{c_{1i} e_{2i} - c_{2i} e_{1i} }}{{c_{1i} d_{2i} - c_{2i} d_{1i} }}; \\ a_{2i}{\prime} = & \frac{{d_{2i} b_{1i} - d_{1i} b_{2i} }}{{d_{1i} c_{2i} - d_{2i} c_{1i} }};\; b_{2i}{\prime} = \frac{{d_{2i} a_{1i} - d_{1i} a_{2i} }}{{d_{1i} c_{2i} - d_{2i} c_{1i} }};\; c_{2i}{\prime} = \frac{{d_{1i} e_{2i} - d_{2i} e_{1i} }}{{d_{1i} c_{2i} - d_{2i} c_{1i} }} \\ \end{aligned}$$

The corresponding section recurrence equations are obtained by sequentially working backwards from the first section of the river to any section:

9 $$\left\{\begin{array}{c}\Delta {Z}_{i+1}=EMT(2i+1, 1)\Delta {Z}_{1}+EMT(2i+1, 2)\Delta {Q}_{1}+EMT(2i+1, 3)\\ \Delta {Q}_{i+1}=EMT(2i+2, 1)\Delta {Z}_{1}+EMT(2i+2, 2)\Delta {Q}_{1}+EMT(2i+2, 3)\end{array}\right.$$

where when *i* = 0

10 $$\left\{\begin{array}{l}EMT(1, 1)=1, \, \, \, EMT(1, 2)=0, \, \, EMT(1, 3)=0\\ EMT(2, 1)=0, \, \, \, EMT(2, 2)=1, \, \, EMT(2, 3)=0\end{array}\right.$$

when *i* > 0,

11 $$\left\{\begin{array}{l}EMT(2i+1, 1)={a}_{1i}{\prime}EMT(2i-1, \, {1})+{b}_{1i}{\prime} EMT(2i, 1)\\ EMT(2i+1, 2)={a}_{1i}{\prime}EMT(2i-1, \, {2})+{b}_{1i}{\prime} EMT(2i, 2)\\ EMT(2i+1, 3)={a}_{1i}{\prime}EMT(2i-1, \, {3})+{b}_{1i}{\prime} EMT(2i, 3)+{c}_{1i}{\prime}\\ EMT(2i+2, 1)={a}_{2i}{\prime}EMT(2i-1, \, {1})+{b}_{2i}{\prime} EMT(2i, 1)\\ EMT(2i+2, 2)={a}_{2i}{\prime}EMT(2i-1, \, {2})+{b}_{2i}{\prime} EMT(2i, 2)\\ EMT(2i+2, 3)={a}_{2i}{\prime}EMT(2i-1, \, {3})+{b}_{2i}{\prime} EMT(2i, 3)+{c}_{2i}{\prime}\end{array}\right.$$

For a river with *m* reaches, the number of cross sections is *m* + 1. Based on Eq. (7), we can get:

12 $$\left\{\begin{array}{l}\Delta {Q}_{m+1}=a\Delta {Z}_{1}+\rightleftarrows b\Delta {Z}_{m+1}+c\\ \Delta {Q}_{1}=d\Delta {Z}_{1}+\rightleftarrows e\Delta {Z}_{m+1}+f\end{array}\right.$$

where

13 $$\begin{aligned} a = & EMT\left( {2m + 2,1} \right) - \frac{{EMT\left( {2m + 1,1} \right)*EMT\left( {2m + 2,2} \right)}}{{EMT\left( {2m + 1,2} \right)}};\;b = \rightleftarrows \frac{{EMT\left( {2m + 2,2} \right)}}{{EMT\left( {2m + 1,2} \right)}}; \\ c = & EMT\left( {2m + 2,3} \right) - \frac{{EMT\left( {2m + 1,3} \right)*EMT\left( {2m + 2,2} \right)}}{{EMT\left( {2m + 1,2} \right)}};\;d = - \frac{{EMT\left( {2m + 1,1} \right)}}{{EMT\left( {2m + 1,2} \right)}}; \\ e = & \rightleftarrows ~\frac{1}{{EMT\left( {2m + 1,2} \right)}};\;f = - \frac{{EMT\left( {2m + 1,3} \right)}}{{EMT\left( {2m + 1,2} \right)}} \\ \end{aligned}$$

According to the above equations, we can see that the flow increment at the first and last cross-sections of the river can be expressed in terms of the increment of water level. Therefore, the corresponding equations using non-increment can be obtained by expanding Eq. (12).

The equations are:

14 $$\left\{\begin{array}{l}{Q}_{m+1}^{t+1}=a{Z}_{1}^{t+1}+ \rightleftarrows b{Z}_{m+1}^{t+1}-a{Z}_{1}^{t}-b{Z}_{m+1}^{t}+{Q}_{m+1}^{t}+c\\ {Q}_{1}^{t+1}\; =d{Z}_{1}^{t+1}+ \rightleftarrows e{Z}_{m+1}^{t+1}-d{Z}_{1}^{t}-e{Z}_{m+1}^{t}+{Q}_{1}^{t}+f\end{array}\right.$$

To solve the hydrodynamic process of a river system, the Saint–Venant equations can be utilized. The existing Saint–Venant equations include a series of nonlinear partial derivative equations^25^. Considering the influence of channel-bed roughness on the hydrodynamic process, the general form of these unsteady hydrodynamic equations can be written as:

15 $$F\left({\boldsymbol{X}},{\boldsymbol{n}},t\right)=0$$

where ${\boldsymbol{X}}$ = the hydraulic state variable, such as discharge, water level or water depth;${\boldsymbol{n}}$ = the river channel-bed roughness, which is taken as a spatiotemporal variable. Note that the conventional form of one-dimensional unsteady hydrodynamic equations is simplified as:

$$F\left({\boldsymbol{X}},t\right)=0$$

given the roughness layer is uniform and varies insignificantly; $t$ = the time.

For the ease of numerical evaluation and implementation, a space–time discretization procedure is carried out for the real-fluid flow in above natural system because the Saint–Venant equations are nonlinear hyperbolic partial differential equations and cannot be solved analytically using the initial conditions^25^. Since the hydraulic state variables are influenced by white noise, the discretized nonlinear hydraulic state equation can be expressed as:

16 $${{\boldsymbol{X}}}_{k}={\varvec{\phi }}_{k}^{\left(1\right)}\left({{\boldsymbol{X}}}_{k-1},{{\boldsymbol{n}}}_{k-1},k-1\right)+{{\varvec{\Gamma}}}_{k}^{(1)}({{\boldsymbol{X}}}_{k-1},{{\boldsymbol{n}}}_{k-1},k-1){{\boldsymbol{W}}}_{k}^{(1)}$$

where ${\varvec{\phi }}_{k}^{\left(1\right)}$ is a nonlinear hydraulic state functional from the state at $k-1$ to the state at $k$, including a series of discrete equations of the hydrodynamic model. The details of the development of these discrete equations by using the finite difference method can be found in^26^; ${\varvec{\Gamma }}_{k}^{(1)}$ is the weighting matrix for the white noise of hydraulic state variables, which is taken as a unit matrix; ${{\boldsymbol{W}}}_{k}^{(1)}$ is a white noise with a mean value that is equal to zero.

In this paper, we aim to improve the simulation accuracy of water level through the prior knowledge of the channel-bed roughness, the roughness is then also discretized as:

17 $${{\boldsymbol{n}}}_{k}={\varvec{\phi }}_{k}^{\left(2\right)}\left({{\boldsymbol{X}}}_{k-1},{{\boldsymbol{n}}}_{k-1},k-1\right)+{\varvec{\Gamma }}_{k}^{(2)}({{\boldsymbol{X}}}_{k-1},{{\boldsymbol{n}}}_{k-1},k-1){{\boldsymbol{W}}}_{k}^{(2)}$$

where ${\varvec{\phi }}_{k}^{\left(2\right)}$ is a roughness function from the state at $k-1$ to the state at $k$; ${\varvec{\Gamma }}_{k}^{(2)}$ is the weight matrix for the white noise of roughness. ${{\boldsymbol{W}}}_{k}^{(2)}$ is a roughness’s white noise with a mean value equal to zero.

The state measurements ${{\boldsymbol{Y}}}_{k}$ (e.g., discharge, water level and water depth) of a river network are then written as:

18 $${{\boldsymbol{Y}}}_{k}={{\boldsymbol{H}}}_{k}{{\boldsymbol{X}}}_{k}+{{\boldsymbol{V}}}_{k}$$

where ${{\boldsymbol{H}}}_{k}$ is the linear measurement function at time $k$; ${{\boldsymbol{V}}}_{k}$ is the measurement noise with a mean value equal to zero. In this paper, state measurement variable, i.e., water level, is selected as a variable for forecasting.

Finally, the hydrodynamic nonlinear dynamic system of a river network considering river roughness as a variable can be obtained by combining Eqs. (2)–(4). The corresponding equations are then written as:

19 $$\left[\begin{array}{c}{{\boldsymbol{X}}}_{k}\\ {{\boldsymbol{n}}}_{k}\end{array}\right]=\left[\begin{array}{c}{\varvec{\phi }}_{k}^{\left(1\right)}\left({{\boldsymbol{X}}}_{k-1},{{\boldsymbol{n}}}_{k-1},k-1\right)\\ {\varvec{\phi }}_{k}^{\left(2\right)}\left({{\boldsymbol{X}}}_{k-1},{{\boldsymbol{n}}}_{k-1},k-1\right)\end{array}\right]+\left[\begin{array}{c}{\varvec{\Gamma }}_{k}^{(1)}({{\boldsymbol{X}}}_{k-1},{{\boldsymbol{n}}}_{k-1},k-1){{\boldsymbol{W}}}_{k}^{(1)}\\ {\varvec{\Gamma }}_{k}^{(2)}({{\boldsymbol{X}}}_{k-1},{{\boldsymbol{n}}}_{k-1},k-1){{\boldsymbol{W}}}_{k}^{(2)}\end{array}\right]$$

20 $${{\boldsymbol{Y}}}_{k}=\left[ {{\boldsymbol{H}}}_{k} 0\right]\left[\begin{array}{c}{{\boldsymbol{X}}}_{k}\\ {{\boldsymbol{n}}}_{k}\end{array}\right]+{{\boldsymbol{V}}}_{k}$$

Note that the nonlinear hydraulic state function ${\varvec{\phi }}_{k}^{\left(1\right)}$ is determined by the numerical discrete form of a river hydrodynamic equation; the measurement function ${{\boldsymbol{H}}}_{k}$ is determined by the measured state variables. In general, the change of roughness between two consecutive time steps is minimal; it thus can be negligible. Consequently, the roughness function ${\varvec{\phi }}_{k}^{\left(2\right)}$ can be expressed:

21 $${\varvec{\phi }}_{k}^{\left(2\right)}:{{\boldsymbol{n}}}_{k}={\boldsymbol{I}}\cdot {{\boldsymbol{n}}}_{k-1}$$

where *I* is a unit matrix.

This paper develops a new data assimilation algorithm with both real-time roughness correction and water level correction based on the extended Kalman filtering algorithm. The river network hydrodynamic system is a nonlinear dynamic system, so it should be linearized to meet the processing requirements of the Kalman filtering algorithm. Given the state estimate ${\widehat{{\boldsymbol{X}}}}_{k-1}$ and ${\widehat{{\boldsymbol{n}}}}_{k-1}$ at time $k-1$, we rewrite the nonlinear hydraulic state equation and river roughness equation by conducting Taylor expansion in terms of ${\widehat{{\boldsymbol{X}}}}_{k-1}$ and ${\widehat{{\boldsymbol{n}}}}_{k-1}$. The linear items are then adopted, which are expressed as:

22 $$\left[\begin{array}{c}{{\boldsymbol{X}}}_{k}\\ {{\boldsymbol{n}}}_{k}\end{array}\right]=\left[\begin{array}{c}{\varvec{\phi }}_{k}^{\left(1\right)}\left({\widehat{{\boldsymbol{X}}}}_{k-1},{\widehat{{\boldsymbol{n}}}}_{k-1},k-1\right)\\ {\varvec{\phi }}_{k}^{\left(2\right)}\left({\widehat{{\boldsymbol{X}}}}_{k-1},{\widehat{{\boldsymbol{n}}}}_{k-1},k-1\right)\end{array}\right]+{\varvec{\Psi }}_{k}\left(\left[\begin{array}{c}{{\boldsymbol{X}}}_{k-1}\\ {{\boldsymbol{n}}}_{k-1}\end{array}\right]-\left[\begin{array}{c}{\widehat{{\boldsymbol{X}}}}_{k-1}\\ {\widehat{{\boldsymbol{n}}}}_{k-1}\end{array}\right]\right)+{\varvec{\Gamma }}_{k}\left[\begin{array}{c}{{\boldsymbol{W}}}_{k}^{(1)}\\ {{\boldsymbol{W}}}_{k}^{(2)}\end{array}\right]$$

where

23 $${\varvec{\Psi }}_{\mathrm{k}}=\left[\begin{array}{cc}\frac{\partial {\varvec{\phi }}_{k}^{\left(1\right)}\left({{\boldsymbol{X}}}_{k-1},{\widehat{{\boldsymbol{n}}}}_{k-1},k-1\right)}{\partial {\widehat{{\boldsymbol{X}}}}_{k-1}}& \frac{\partial {\varvec{\phi }}_{k}^{\left(1\right)}\left({\widehat{{\boldsymbol{X}}}}_{k-1},{{\boldsymbol{n}}}_{k-1},k-1\right)}{\partial {\widehat{{\boldsymbol{n}}}}_{k-1}}\\ \frac{\partial {\varvec{\phi }}_{k}^{\left(2\right)}\left({{\boldsymbol{X}}}_{k-1},{\widehat{{\boldsymbol{n}}}}_{k-1},k-1\right)}{\partial {\widehat{{\boldsymbol{X}}}}_{k-1}}& \frac{\partial {\varvec{\phi }}_{k}^{\left(2\right)}\left({\widehat{{\boldsymbol{X}}}}_{k-1},{{\boldsymbol{n}}}_{k-1},k-1\right)}{\partial {\widehat{{\boldsymbol{n}}}}_{k-1}}\end{array}\right]$$

is tangent state operator, and

24 $${\varvec{\Gamma }}_{k}=\left[\begin{array}{cc}{\varvec{\Gamma }}_{k}^{\left(1\right)}\left({\widehat{{\boldsymbol{X}}}}_{k-1},{\widehat{{\boldsymbol{n}}}}_{k-1},k-1\right)& 0\\ 0& {\varvec{\Gamma }}_{k}^{\left(2\right)}\left({\widehat{{\boldsymbol{X}}}}_{k-1},{\widehat{{\boldsymbol{n}}}}_{k-1},k-1\right)\end{array}\right]$$

is noise weight matrix, which is usually taken as unit matrix ***I***.

In this paper, the new data assimilation scheme using the extended Kalman filtering technology is used for water level forecasting of river networks considering the simultaneous real-time correction of roughness and water level.

The corresponding real-time correction algorithms are as follows:

1. The first step is to obtain the simulation values of water level and roughness for forecasting:
  25 $$\left[\begin{array}{c}{\widehat{{\boldsymbol{X}}}}_{k/k-1}\\ {\widehat{{\boldsymbol{n}}}}_{k/k-1}\end{array}\right]=\left[\begin{array}{c}{\varvec{\phi }}_{k}^{\left(1\right)}\left({\widehat{{\boldsymbol{X}}}}_{k-1},{\widehat{{\boldsymbol{n}}}}_{k-1},k-1\right)\\ {\varvec{\phi }}_{k}^{\left(2\right)}\left({\widehat{{\boldsymbol{X}}}}_{k-1},{\widehat{{\boldsymbol{n}}}}_{k-1},k-1\right)\end{array}\right]$$
2. Then the prediction error variance matrix is obtained:
  26 $${{\boldsymbol{P}}}_{k/k-1}={{\boldsymbol{\Psi}}}_{\mathrm{k}}{{\boldsymbol{P}}}_{k-1}{{\boldsymbol{\Psi}}}_{k}^{T}+{\varvec{\Gamma }}_{k}{{\boldsymbol{Q}}}_{k}{\varvec{\Gamma }}_{k}^{T}$$
  where
  27 $${{\boldsymbol{Q}}}_{k}=\left[\begin{array}{cc}{{\boldsymbol{Q}}}_{k}^{\left(1\right)}& 0\\ 0& {{\boldsymbol{Q}}}_{k}^{\left(2\right)}\end{array}\right]$$
  is white noise covariance matrix; ${{\boldsymbol{Q}}}_{k}^{\left(1\right)}$ is the covariance matrix for the white noise of water level; ${{\boldsymbol{Q}}}_{k}^{\left(2\right)}$ is covariance matrix for the white noise of roughness.
3. Gain matrix is also obtained as:
  28 $${{\boldsymbol{K}}}_{k}={{\boldsymbol{P}}}_{k/k-1}{\left[\begin{array}{cc}{{\boldsymbol{H}}}_{k}& 0\end{array}\right]}^{T}{\left(\left[\begin{array}{cc}{{\boldsymbol{H}}}_{k}& 0\end{array}\right]{{\boldsymbol{P}}}_{k/k-1}{\left[\begin{array}{cc}{{\boldsymbol{H}}}_{k}& 0\end{array}\right]}^{T}+{{\boldsymbol{R}}}_{k}\right)}^{-1}$$
  where ${{\boldsymbol{R}}}_{k}$ is measurement noise covariance matrix.
4. The water level and roughness are then updated as:
  29 $$\left[\begin{array}{c}{\widehat{{\boldsymbol{X}}}}_{k}\\ {\widehat{{\boldsymbol{n}}}}_{k}\end{array}\right]=\left[\begin{array}{c}{\widehat{{\boldsymbol{X}}}}_{k/k-1}\\ {\widehat{{\boldsymbol{n}}}}_{k/k-1}\end{array}\right]+{{\boldsymbol{K}}}_{k}\left({{\boldsymbol{Y}}}_{k}-\left[\begin{array}{cc}{{\boldsymbol{H}}}_{k}& 0\end{array}\right]\left[\begin{array}{c}{\widehat{{\boldsymbol{X}}}}_{k/k-1}\\ {\widehat{{\boldsymbol{n}}}}_{k/k-1}\end{array}\right]\right)$$
5. Finally, the correction error variance matrix is calculated:
  30 $${{\boldsymbol{P}}}_{k}=\left({\boldsymbol{I}}-{{\boldsymbol{K}}}_{k}\left[\begin{array}{cc}{{\boldsymbol{H}}}_{k}& 0\end{array}\right]\right){{\boldsymbol{P}}}_{k/k-1}$$

If the state vector ***X*** contains *m* water level or flow variables, the roughness vector ***n*** contains *n* roughness parameters, and the measurement vector ***Y*** contains *s* observed variables, then the corresponding quantities in the algorithm are elaborated as follows:

1. The filter error covariance matrix at time *k − 1* can be denoted as
  31 $${{\boldsymbol{P}}}_{k-1}=\left[\begin{array}{cc}\underset{m\times m}{{{\boldsymbol{P}}}_{11}^{k-1}}& \underset{m\times n}{{{\boldsymbol{P}}}_{12}^{k-1}}\\ \underset{n\times m}{{{\boldsymbol{P}}}_{21}^{k-1}}& \underset{n\times n}{{{\boldsymbol{P}}}_{22}^{k-1}}\end{array}\right]\stackrel{simplified as}{=}\left[\begin{array}{cc}\underset{m\times m}{{{\boldsymbol{P}}}_{11}}& \underset{m\times n}{{{\boldsymbol{P}}}_{12}}\\ \underset{n\times m}{{{\boldsymbol{P}}}_{21}}& \underset{n\times n}{{{\boldsymbol{P}}}_{22}}\end{array}\right]$$
2. The tangential state operator can be denoted as:
  $${\varvec{\Psi }}_{k}=\left[\begin{array}{cc}\frac{\partial {\varvec{\phi }}_{k}^{\left(1\right)}\left({{\boldsymbol{X}}}_{k-1},{\widehat{{\boldsymbol{n}}}}_{k-1},k-1\right)}{\partial {\widehat{{\boldsymbol{X}}}}_{k-1}}& \frac{\partial {\varvec{\phi }}_{k}^{\left(1\right)}\left({\widehat{{\boldsymbol{X}}}}_{k-1},{{\boldsymbol{n}}}_{k-1},k-1\right)}{\partial {\widehat{{\boldsymbol{n}}}}_{k-1}}\\ \frac{\partial {\varvec{\phi }}_{k}^{\left(2\right)}\left({{\boldsymbol{X}}}_{k-1},{\widehat{{\boldsymbol{n}}}}_{k-1},k-1\right)}{\partial {\widehat{{\boldsymbol{X}}}}_{k-1}}& \frac{\partial {\varvec{\phi }}_{k}^{\left(2\right)}\left({\widehat{{\boldsymbol{X}}}}_{k-1},{{\boldsymbol{n}}}_{k-1},k-1\right)}{\partial {\widehat{{\boldsymbol{n}}}}_{k-1}}\end{array}\right]$$
  32 $$=\left[\begin{array}{cc}\underset{m\times m}{{\varvec{\Psi }}_{11}^{k}}& \underset{m\times n}{{\varvec{\Psi }}_{12}^{k}}\\ \underset{n\times m}{{\varvec{\Psi }}_{21}^{k}}& \underset{n\times n}{{\varvec{\Psi }}_{22}^{k}}\end{array}\right]\stackrel{simplified as}{=}\left[\begin{array}{cc}\underset{m\times m}{{\varvec{\Psi }}_{11}}& \underset{m\times n}{{\varvec{\Psi }}_{12}}\\ \underset{n\times m}{{\varvec{\Psi }}_{21}}& \underset{n\times n}{{\varvec{\Psi }}_{22}}\end{array}\right]$$
3. The dynamic noise covariance can be denoted as
  33 $${{\boldsymbol{Q}}}_{k}=\left[\begin{array}{cc}\underset{m\times m}{{{\boldsymbol{Q}}}_{11}^{k}}& 0\\ 0& \underset{n\times n}{{{\boldsymbol{Q}}}_{22}^{k}}\end{array}\right]\stackrel{simplified as}{=}\left[\begin{array}{cc}\underset{m\times m}{{{\boldsymbol{Q}}}_{11}}& 0\\ 0& \underset{n\times n}{{{\boldsymbol{Q}}}_{22}}\end{array}\right]$$
4. The observation noise covariance can be denoted as:
  34 $${{\boldsymbol{R}}}_{k}=\underset{s\times s}{{{\boldsymbol{R}}}_{k}}$$
5. predicted error variance matrix using Eqs. (17) to (20), can be obtained as:
  $${{\boldsymbol{P}}}_{k/k-1}={\varvec{\Psi }}_{k}{{\boldsymbol{P}}}_{k-1}{\varvec{\Psi }}_{k}^{T}+{\varvec{\Gamma }}_{k}{{\boldsymbol{Q}}}_{k}{\varvec{\Gamma }}_{k}^{T}=\left[\begin{array}{cc}({\varvec{\Psi }}_{11}{{\boldsymbol{P}}}_{11}+{\varvec{\Psi }}_{12}{{\boldsymbol{P}}}_{21}){\varvec{\Psi }}_{11}^{T}+({\varvec{\Psi }}_{11}{{\boldsymbol{P}}}_{12}+{\varvec{\Psi }}_{12}{{\boldsymbol{P}}}_{22}){\varvec{\Psi }}_{12}^{T}+{{\boldsymbol{Q}}}_{11}& ({\varvec{\Psi }}_{11}{{\boldsymbol{P}}}_{11}+{\varvec{\Psi }}_{12}{{\boldsymbol{P}}}_{21}){\varvec{\Psi }}_{21}^{T}+({\varvec{\Psi }}_{11}{{\boldsymbol{P}}}_{12}+{\varvec{\Psi }}_{12}{{\boldsymbol{P}}}_{22}){\varvec{\Psi }}_{22}^{T}\\ ({\varvec{\Psi }}_{21}{{\boldsymbol{P}}}_{11}+{\varvec{\Psi }}_{22}{{\boldsymbol{P}}}_{21}){\varvec{\Psi }}_{11}^{T}+({\varvec{\Psi }}_{21}{{\boldsymbol{P}}}_{12}+{\varvec{\Psi }}_{22}{{\boldsymbol{P}}}_{22}){\varvec{\Psi }}_{12}^{T}& ({\varvec{\Psi }}_{21}{{\boldsymbol{P}}}_{11}+{\varvec{\Psi }}_{22}{{\boldsymbol{P}}}_{21}){\varvec{\Psi }}_{21}^{T}+({\varvec{\Psi }}_{21}{{\boldsymbol{P}}}_{12}+{\varvec{\Psi }}_{22}{{\boldsymbol{P}}}_{22}){\varvec{\Psi }}_{22}^{T}+{{\boldsymbol{Q}}}_{22}\end{array}\right]$$
  35 $$\stackrel{simplified as }{=} \, \left[\begin{array}{cc}\underset{m\times m}{{{\boldsymbol{P}}}_{11}^{k/k-1}}& \underset{m\times n}{{{\boldsymbol{P}}}_{12}^{k/k-1}}\\ \underset{n\times m}{{{\boldsymbol{P}}}_{21}^{k/k-1}}& \underset{n\times n}{{{\boldsymbol{P}}}_{22}^{k/k-1}}\end{array}\right]$$
6. Gain matrix is obtained as:
  36 $${{\boldsymbol{K}}}_{k}={{\boldsymbol{P}}}_{k/k-1}{\left[\begin{array}{cc}{{\boldsymbol{H}}}_{k}& 0\end{array}\right]}^{T}{\left[\left[\begin{array}{cc}{{\boldsymbol{H}}}_{k}& 0\end{array}\right]{{\boldsymbol{P}}}_{k/k-1}{\left[\begin{array}{cc}{{\boldsymbol{H}}}_{k}& 0\end{array}\right]}^{T}+{{\boldsymbol{R}}}_{k}\right]}^{-1}\left[\begin{array}{c}\underset{m\times s}{{{\boldsymbol{P}}}_{11}^{k/k-1}{{\boldsymbol{H}}}_{k}^{T}}\\ \underset{n\times s}{{{\boldsymbol{P}}}_{21}^{k/k-1}{{\boldsymbol{H}}}_{k}^{T}}\end{array}\right]{\left[\underset{s\times s}{{{\boldsymbol{H}}}_{k}{{\boldsymbol{P}}}_{11}^{k/k-1}{{\boldsymbol{H}}}_{k}^{T}}+\underset{s\times s}{{{\boldsymbol{R}}}_{k}}\right]}^{-1}=\left[\begin{array}{c}\underset{m\times s}{{{\boldsymbol{P}}}_{11}^{k/k-1}{{\boldsymbol{H}}}_{k}^{T}{\left[{{\boldsymbol{H}}}_{k}{{\boldsymbol{P}}}_{11}^{k/k-1}{{\boldsymbol{H}}}_{k}^{T}+{{\boldsymbol{R}}}_{k}\right]}^{-1}}\\ \underset{n\times s}{{{\boldsymbol{P}}}_{21}^{k/k-1}{{\boldsymbol{H}}}_{k}^{T}{\left[{{\boldsymbol{H}}}_{k}{{\boldsymbol{P}}}_{11}^{k/k-1}{{\boldsymbol{H}}}_{k}^{T}+{{\boldsymbol{R}}}_{k}\right]}^{-1}}\end{array}\right]$$
7. The filtering error variance matrix at time *k* can be denoted as:
  $${{\boldsymbol{P}}}_{k}=({\boldsymbol{I}}-{{\boldsymbol{K}}}_{k}\left[\begin{array}{cc}{{\boldsymbol{H}}}_{k}& 0\end{array}\right]){{\boldsymbol{P}}}_{k/k-1}$$
  $$=\left[\begin{array}{cc}\underset{m\times m}{{\boldsymbol{I}}-{{\boldsymbol{P}}}_{11}^{k/k-1}{{\boldsymbol{H}}}_{k}^{T}{\left[{{\boldsymbol{H}}}_{k}{{\boldsymbol{P}}}_{11}^{k/k-1}{{\boldsymbol{H}}}_{k}^{T}+{{\boldsymbol{R}}}_{k}\right]}^{-1}{{\boldsymbol{H}}}_{k}}& 0\\ \underset{n\times m}{-{{\boldsymbol{P}}}_{21}^{k/k-1}{{\boldsymbol{H}}}_{k}^{T}{\left[{{\boldsymbol{H}}}_{k}{{\boldsymbol{P}}}_{11}^{k/k-1}{{\boldsymbol{H}}}_{k}^{T}+{{\boldsymbol{R}}}_{k}\right]}^{-1}{{\boldsymbol{H}}}_{k}}& \underset{n\times n}{{\boldsymbol{I}}}\end{array}\right] \, \left[\begin{array}{cc}\underset{m\times m}{{{\boldsymbol{P}}}_{11}^{k/k-1}}& \underset{m\times n}{{{\boldsymbol{P}}}_{12}^{k/k-1}}\\ \underset{n\times m}{{{\boldsymbol{P}}}_{21}^{k/k-1}}& \underset{n\times n}{{{\boldsymbol{P}}}_{22}^{k/k-1}}\end{array}\right]$$
  37 $$\begin{aligned} {{\boldsymbol{P}}}_{k}& =({\boldsymbol{I}}-{{\boldsymbol{K}}}_{k}\left[\begin{array}{cc}{{\boldsymbol{H}}}_{k}& 0\end{array}\right]){{\boldsymbol{P}}}_{k/k-1}\\ & =\left[\begin{array}{cc}\underset{m\times m}{{\boldsymbol{I}}-{{\boldsymbol{P}}}_{11}^{k/k-1}{{\boldsymbol{H}}}_{k}^{T}{\left[{{\boldsymbol{H}}}_{k}{{\boldsymbol{P}}}_{11}^{k/k-1}{{\boldsymbol{H}}}_{k}^{T}+{{\boldsymbol{R}}}_{k}\right]}^{-1}{{\boldsymbol{H}}}_{k}}& 0\\ \underset{n\times m}{-{{\boldsymbol{P}}}_{21}^{k/k-1}{{\boldsymbol{H}}}_{k}^{T}{\left[{{\boldsymbol{H}}}_{k}{{\boldsymbol{P}}}_{11}^{k/k-1}{{\boldsymbol{H}}}_{k}^{T}+{{\boldsymbol{R}}}_{k}\right]}^{-1}{{\boldsymbol{H}}}_{k}}& \underset{n\times n}{{\boldsymbol{I}}}\end{array}\right] \, \left[\begin{array}{cc}\underset{m\times m}{{{\boldsymbol{P}}}_{11}^{k/k-1}}& \underset{m\times n}{{{\boldsymbol{P}}}_{12}^{k/k-1}}\\ \underset{n\times m}{{{\boldsymbol{P}}}_{21}^{k/k-1}}& \underset{n\times n}{{{\boldsymbol{P}}}_{22}^{k/k-1}}\end{array}\right]\\& =\left[\begin{array}{cc}\underset{m\times m}{({\boldsymbol{I}}-{{\boldsymbol{P}}}_{11}^{k/k-1}{{\boldsymbol{H}}}_{k}^{T}{\left[{{\boldsymbol{H}}}_{k}{{\boldsymbol{P}}}_{11}^{k/k-1}{{\boldsymbol{H}}}_{k}^{T}+{{\boldsymbol{R}}}_{k}\right]}^{-1}{{\boldsymbol{H}}}_{k}){{\boldsymbol{P}}}_{11}^{k/k-1}}& \underset{m\times n}{({\boldsymbol{I}}-{{\boldsymbol{P}}}_{11}^{k/k-1}{{\boldsymbol{H}}}_{k}^{T}{\left[{{\boldsymbol{H}}}_{k}{{\boldsymbol{P}}}_{11}^{k/k-1}{{\boldsymbol{H}}}_{k}^{T}+{{\boldsymbol{R}}}_{k}\right]}^{-1}{{\boldsymbol{H}}}_{k}){{\boldsymbol{P}}}_{12}^{k/k-1}}\\ \underset{n\times m}{-{{\boldsymbol{P}}}_{21}^{k/k-1}{{\boldsymbol{H}}}_{k}^{T}{\left[{{\boldsymbol{H}}}_{k}{{\boldsymbol{P}}}_{11}^{k/k-1}{{\boldsymbol{H}}}_{k}^{T}+{{\boldsymbol{R}}}_{k}\right]}^{-1}{{\boldsymbol{H}}}_{k}{{\boldsymbol{P}}}_{11}^{k/k-1}+{{\boldsymbol{P}}}_{21}^{k/k-1}}& \underset{n\times n}{-{{\boldsymbol{P}}}_{21}^{k/k-1}{{\boldsymbol{H}}}_{k}^{T}{\left[{{\boldsymbol{H}}}_{k}{{\boldsymbol{P}}}_{11}^{k/k-1}{{\boldsymbol{H}}}_{k}^{T}+{{\boldsymbol{R}}}_{k}\right]}^{-1}{{\boldsymbol{H}}}_{k}{{\boldsymbol{P}}}_{12}^{k/k-1}+{{\boldsymbol{P}}}_{22}^{k/k-1}}\end{array}\right] \end{aligned}$$

The above data filtering process is cycled continuously to update the simulated water level, discharge and river-bed roughness values for reach simulation moment with the elapsed time. If the correction error variance matrix is less than a certain critical value, the data assimilation process can be ended to improve the efficiency of the river network hydrodynamic model calculation. In practice, the data assimilation process should be re-started if the simulated water level values deviate from the measured values by a certain degree after the end of data assimilation.

The above data filtering process with simultaneous consideration of water level and roughness correction with the recurrence of time is then compared to that without real-time roughness correction.

## Experimental evaluation

### Case description

**Figure 1:**
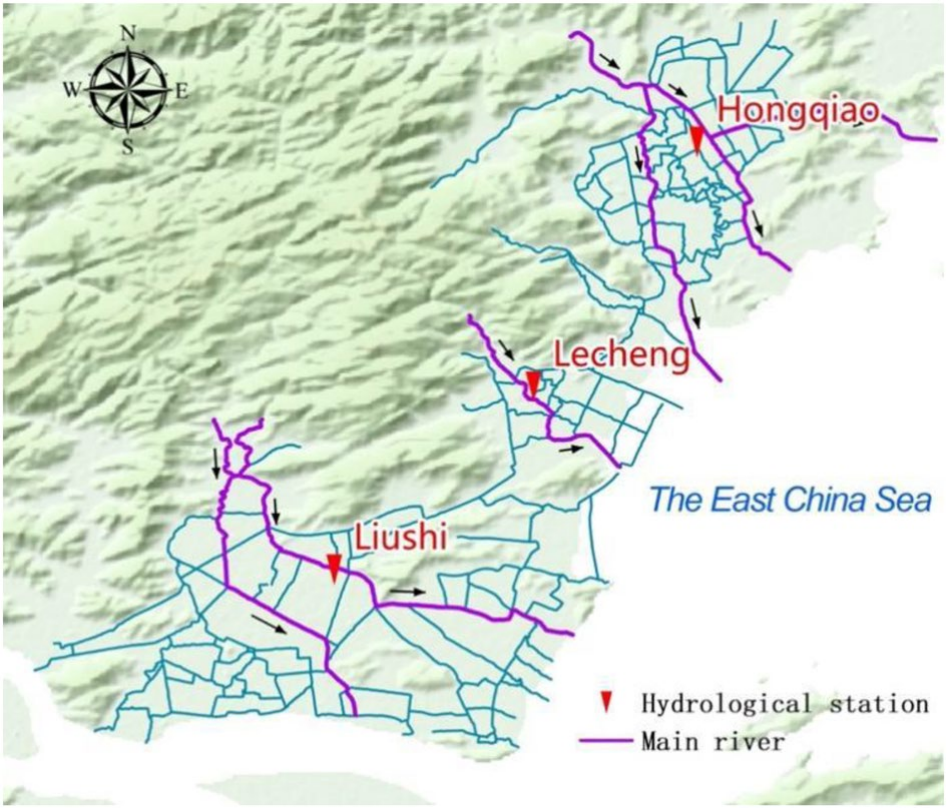
The schematic diagram of the Le Liu Hong plain river network.

A real river network named Le Liu Hong plain river network, with an area of 325 km^2^ in China, was used to evaluate the effectiveness of the proposed data assimilation model. It is located in the southeast of Zhejiang Province, China. The river mouth is located on the coast of mainland China in the East China Sea. This river network has three sub-systems: Hongqiao, Lecheng and Liushi, according to topography and geomorphology, as demonstrated in Fig. 1. Each sub-system has a water level measuring station, i.e., Hongqiao station, Lecheng station and Liushi station. The statistics show that the hydrodynamic model of this Le Liu Hong plain river network includes 398 rivers, 304 nodes and a total of 1962 cross-sections.

Key foundational data was collected from a river network to construct the data assimilation system in this study. The measurement approach included establishing a cross-section every 200 m on average along the river network, with a more concentrated measurement approach for sections that suddenly narrowed or widened. Specific structures, such as sluices and dams, were individually measured to determine their geometric dimensions. Three strategically placed water level stations provided spatially distributed data, located in the central parts of three sub-areas of the plain river network, each approximately 12 km apart. These stations collected water level data every 5 min, ensuring a frequent and consistent stream of observational data. The study utilized data gathered during the 2019 Lekima Typhoon flood event, collected from 8 AM on August 7, 2019, to 8 AM on August 12, 2019, thus offering a continuous, high-frequency dataset over a 5-day period. This data set is directly stored in our computer systems, enabling immediate access for comparison with our model's calculated results. The stored data is processed by our data assimilation system running the proposed algorithm to execute the simultaneous correction of roughness and water level. The Windows 10 computer used for the analysis was equipped with an Intel Core i7-8700 K processor with a clock speed of 3.2 GHz and DDR4 RAM of 16.0 GB.

### Parameter setting and evaluation metrics

The initial channel-bed roughness for each section is set as 0.02. As the roughness is taken as a spatiotemporal variable, the real-time roughness values vary with the model running at different river sections. As the observation noise level $\delta$ could impact the data assimilation results, a series of $\delta$ values, i.e., 0, 0.0001, 0.0005, 0.001, 0.005, 0.01, and 0.05 m are used for understanding the influence of observation noise level on water level forecasting. Note that, in practice, the plain river network water level measurement error is normally between 0.001 and 0.01 m; during typhoons, it will be slightly larger. The corresponding relative observation noise values are less than 2% of water depth values.

To compare the forecasting performance of model configurations with roughness correction and without, the time for the algorithm to reach simulation stability, i.e., filtering time, is used as another evaluation metric. In addition, the root mean square error (RMSE) of the forecasting values compared to the measurement values at the start of data assimilation until the end of the water level simulation is taken as another evaluation metric. The calculation of RMSE can be written as:

38 $$RMSE = \sqrt {\frac{{\mathop \sum \nolimits_{i = 1}^{n} \left( {{\boldsymbol{S}}_{i} - {\boldsymbol{M}}_{i} } \right)^{2} }}{N}}$$

where ${\boldsymbol{M}}_{i}$ are the measurements, ${\boldsymbol{S}}_{i}$ are predicted values, and ${\boldsymbol{N}}$ is the number of the measurements available for analysis.

The simulation period is from 8:00 on August 7, 2019, to 8:00 on August 12, 2019 (5 days in total), assuming data assimilation from 0:00 on August 9, 2019. The filtering is ended if the elements on the diagonal of the water level correction error variance matrix are less than 0.001^2^ m. The data assimilation algorithm based on the EKF with only water level correction is used as a reference algorithm.

### Results and discussion

**Figure 2:**
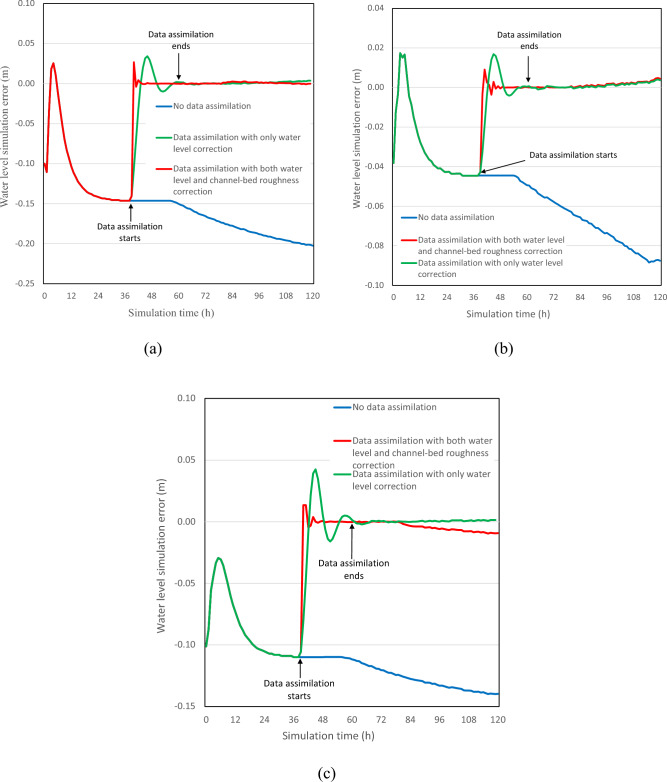
Water level simulation errors produced by different data assimilation schemes at (**a**) Hongqiao station, (**b**) Lecheng station, (**c**) Liushi station. The blue line represents that the data is not treated by data assimilation; the green line represents the treatment by EKF with only water lever correction; the red line represents the treatment by EKF with both water level and channel-bed roughness correction.

Figure 2 presents the water level simulation errors compared to the measured values when the observation noise level value is equal to 0.005 m. Similar results are obtained for other observation noise level values (i.e., 0, 0.0001, 0.0005, 0.001, 0.01, and 0.05 m). The data assimilation treatment is rather effective in reducing simulation errors, as the errors approach zero after the end of data assimilation at both measurement stations. However, the water level simulation errors increase with simulation time if no data assimilation treatment is performed (blue line in Fig. 2). Compared to the data assimilation with only water level correction (i.e., no channel-bed roughness correction), the one with both water level and channel-bed roughness correction produces a remarkable improvement in water level forecasting capacity. Figure 2 demonstrates that the filtering time until the stabilization of simulation error is much shorter regardless of the observation noise level for the model with roughness correction. The filtering time reduces significantly by 44–63%, as shown in Table 1. This relative change percentage is calculated by initially deducting the filtering time in the case with both water level and channel-bed roughness correction from the filtering time in the case with only water level correction, then dividing the result by the filtering time in the case with both water level and channel-bed roughness correction, and then finally multiplying by 100% to express in terms of percentage, as illustrated in Eq. (18). In addition, a large variation amplitude of water level forecasting errors is also observed during the data assimilation for the model without roughness correction.

39 $$RMSE = \frac{{\left( {t_{both} - t_{water\_correction} } \right)}}{{t_{both} }} \times 100\%$$

where $t_{both}$ is the filtering time in the case with both water level and channel-bed roughness correction, $t_{water\_correction}$ is the filtering time in the case with only water level correction.

**Table 1:** Filtering time at different observation noise levels for two data assimilation schemes.

Data assimilation algorithm	Observation noise level δ (m)						
	0.0	0.0001	0.0005	0.001	0.005	0.01	0.05
With only water level correction	Instant	Instant	2 h	4 h	14 h	25 h	*
With both water level and channel-bed roughness correction	Instant	Instant	1 h	1.5 h	7 h	14 h	22 h
Decreasing percentage	–	–	50%	63%	50%	44%	–

*Means non-convergence.

**Figure 3:**
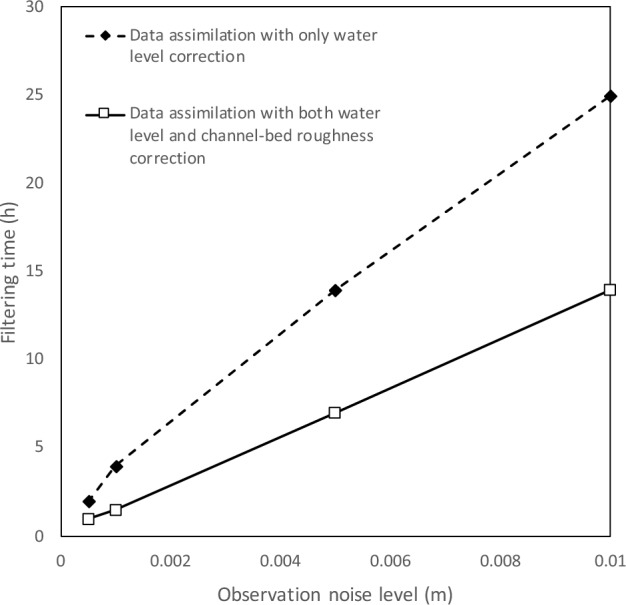
Influence of observation noise level on the filtering time for the Le Liu Hong plain river network.

**Figure 4:**
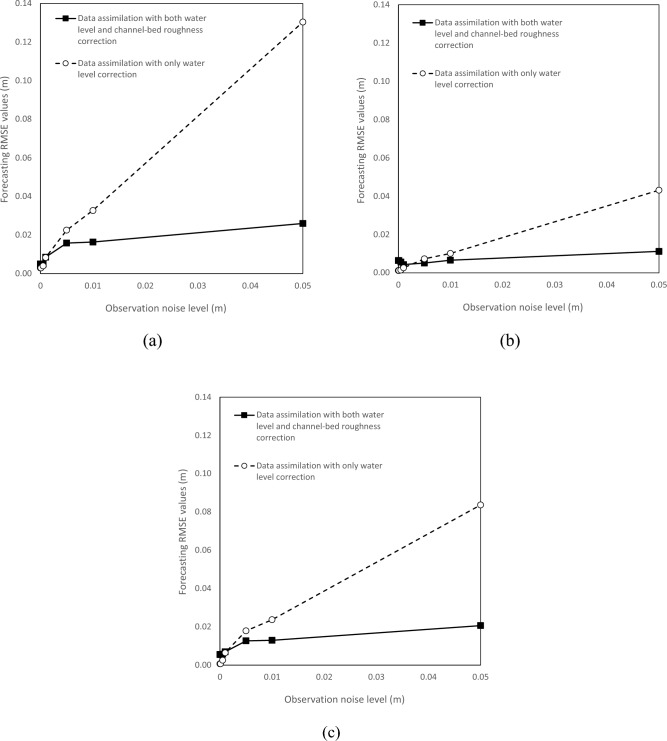
Water level forecasting error evaluation by RMSE with different observation noise levels for both models at (**a**) Hongqiao station, (**b**) Lecheng station, (**c**) Liushi station.

In addition, the filtering time approximately linearly increases with the increasing observation noise level value for both data assimilation schemes (Fig. 3), while the increasing rate is much lower for the data assimilation model with both water level and channel-bed roughness correction compared to that without roughness correction. A similar observation is found for the water level forecasting error evaluation as RMSE values for the data assimilation model with only water level correction remarkably increase with the observation noise level, while RMSE values for the other model increase initially and stabilize at small values afterward at all the stations (Table 2; Fig. 4). For example, the water level RMSE values are from minimal up to 0.13 and 0.026 at Hongqiao station for the model with roughness correction and without, respectively. The RMSE decreases up to 80% at Hongqiao station when the observation noise level is 0.05. Thus, the forecasting accuracy of the data assimilation model with roughness correction is insensitive to the observation noise within a normal range (i.e., less than 0.01 m).

**Table 2:** Impact of observation noise level on the data assimilation performance regarding forecasting RMSE.

Data assimilation algorithm	Observation noise level δ (m)						
	0.0	0.0001	0.0005	0.001	0.005	0.01	0.05
Hongqiao station							
None	0.173	–	–	–	–	–	–
With only water level correction	0.003	0.003	0.004	0.008	0.023	0.033	0.130
With both water level and channel-bed roughness correction	0.005	0.005	0.005	0.008	0.016	0.016	0.026
Lecheng station							
None	0.065	–	–	–	–	–	–
With only water level correction	0.001	0.001	0.002	0.003	0.007	0.010	0.043
With both water level and channel-bed roughness correction	0.006	0.006	0.006	0.004	0.005	0.007	0.011
Liushi station							
None	0.124	–	–	–	–	–	–
With only water level correction	0.001	0.001	0.003	0.007	0.018	0.024	0.084
With both water level and channel-bed roughness correction	0.006	0.006	0.006	0.007	0.013	0.013	0.021

The above observations indicate that the channel-bed roughness used as prior knowledge to be updated with the elapsed time is useful to restrict filtering cost and reduce forecasting errors. For a highly accurate measuring water level data (e.g., a controlled observation noise level value of 0.005 m) in practice, the model with both water level and bed roughness corrections could better predict after 7 h data assimilation, causing a minor forecasting error.

## Parameter analysis

### Description of river network

**Figure 5:**
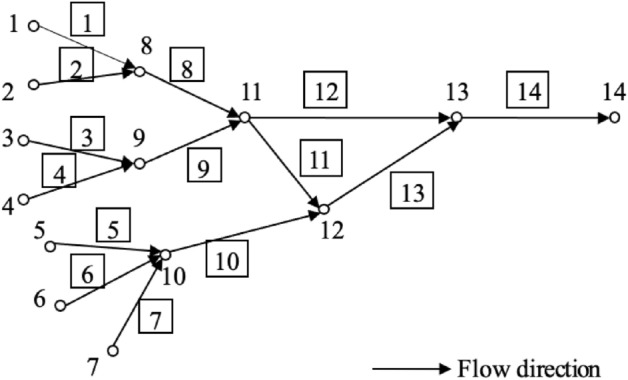
The schematic diagram of the simulated water river network.

The above experimental evaluation on a real river network shows the high sensitivity of filtering time and low sensitivity of our model’s forecasting accuracy to the observation noise level. Whether this result is impacted by the other critical parameter (i.e., initial channel-bed roughness) and how this initial roughness influences data filtering and the forecasting performance are analyzed in this section. To simplify the calculation and reduce computational cost, a case study on a simulated river network with unsteady-state flows is carried out in this section. Note that this river network is referred from the literature^27^ and illustrated in Fig. 5.

### Parameter setting and boundary conditions

The observation noise level variable has values of 0.0, 0.0001, 0.0005, 0.001, 0.005, 0.01 and 0.05 m. The initial value of channel-bed roughness at each river section is the same and is set as 0.020, 0.023, or 0.026. The true roughness of river section 1–8, 10–12, and 14 is 0.023. The remaining river sections 9 and 13 have a true roughness of 0.026 and 0.020, respectively. Table 3 shows the parameter combination scheme of initial roughness and observation noise level.

**Table 3:** The test combination scheme.

Initial roughness	Observation noise level *δ*/m						
	0.0	0.0001	0.0005	0.001	0.005	0.01	0.05
0.020	√	√	√	√	√	√	√
0.023	√	√	√	√	√	√	√
0.026	√	√	√	√	√	√	√

√ means the simulation is successful.

**Figure 6:**
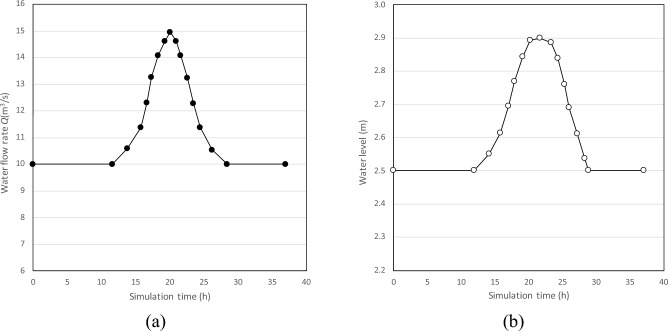
Boundary condition for the (**a**) upstream entrance and (**b**) exit.

To simplify the calculation, the boundary conditions at each entrance are assumed to be the same. The water flow rate from Node 1 to Node 7 was inputted into the model as the boundary condition of the upstream entrance (Fig. 6a). The water level at the exit (i.e., Node 14) was set as the downstream boundary condition (Fig. 6b). Water level measuring points are arranged at Nodes 9 and 13, which are recorded as Station 1 and Station 2.

The RMSE of water level forecasting (hereinafter referred to as "water level RMSE") and the RMSE of channel-bed roughness (hereinafter referred to as "roughness RMSE") are selected as a model performance evaluation index. The impact of observation noise level on the filtering time with different scenarios of initial channel-bed roughness is also analyzed.

### Results and discussion

**Figure 7:**
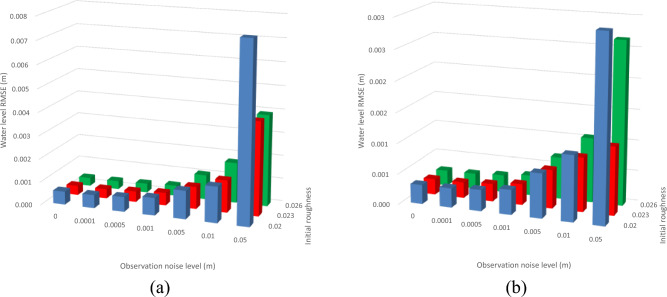
Impact of observation noise level and initial roughness on water level RMSE at (**a**) Station 1 and (**b**) Station 2.

**Figure 8:**
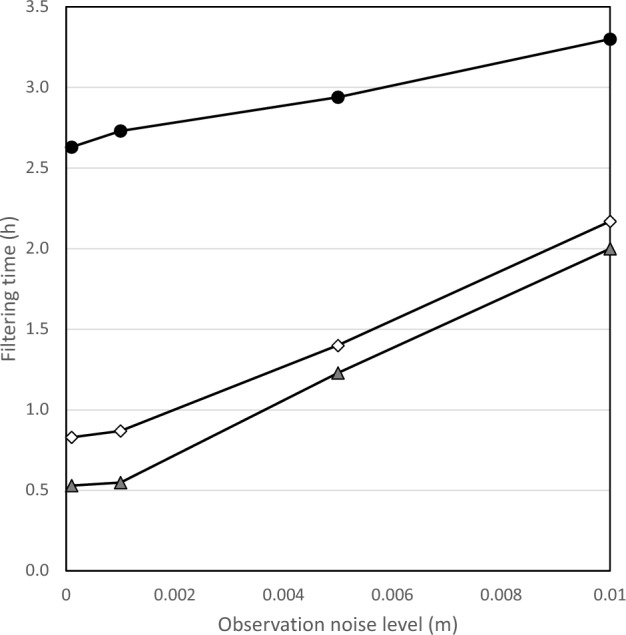
Influence of observation noise level on the filtering time for the simulated river network.

Figure 7 shows that water level RMSE significantly increases with observation noise level regardless of initial channel-bed roughness at two measurement stations. This observation indicates that the impact of observation noise level on the data assimilation and forecasting accuracy, found in “Experimental evaluation”, is not interfered with by the setting of the initial channel-bed roughness. Also, the simulated river network demonstrates that filtering time is highly sensitive to observation noise level regardless of initial channel-bed roughness, as shown in Fig. 8. Consequently, the careful measurement of water level in practice should be taken to achieve efficient data assimilation and water level forecasting. For a normal range of observation noise level in practice (i.e., less than 0.01 m), our data assimilation model with both channel-bed roughness correction and water level correction could maintain a low water level RMSE and a short filtering time; thus, it has a good capacity of water level forecasting.

Interestingly, the water level RMSE values in the simulated river network are much lower than that in the practice river network (i.e., Le Liu Hong plain river network). As shown in Table 2, the water level RMSE at different measurement stations for the Le Liu Hong plain river network ranges from 0.005 to 0.026 m, while the water level RMSE for the simulated river network is generally less than 0.005 m, as presented in Fig. 7. This is reasonable as the practice river network is more complex than the simulated one.

**Figure 9:**
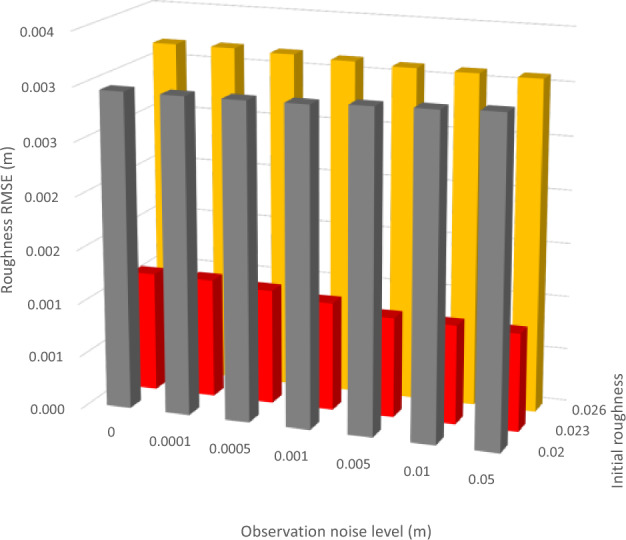
Impact of observation noise level and initial roughness on roughness RMSE.

Figure 7 also shows that the data assimilation treatment with the initial roughness value of 0.023 produces the minimum water level RMSE values compared to the other two initial roughness values (i.e., 0.020 and 0.026). This result is also consistent with the variation of the roughness RMSE with different initial roughness values at different observation noise levels, as shown in Fig. 9. Given that most river sections have true roughness of 0.023, and only one river section has true roughness of 0.020 (i.e., section 13) and 0.026 (i.e., section 9), indicating that the closer the initial channel-bed roughness value is to the true roughness value, the more accurate the forecasting result is. A trial-and-error testing can be conducted in the river network to estimate the optimum channel-bed roughness value as an input to the data assimilation model. Figure 9 also shows that the roughness RMSE is generally insensitive to the change of observation noise level. This is expected as the channel-bed roughness is determined by the river bed nature rather than the water level measurements.

**Figure 10:**
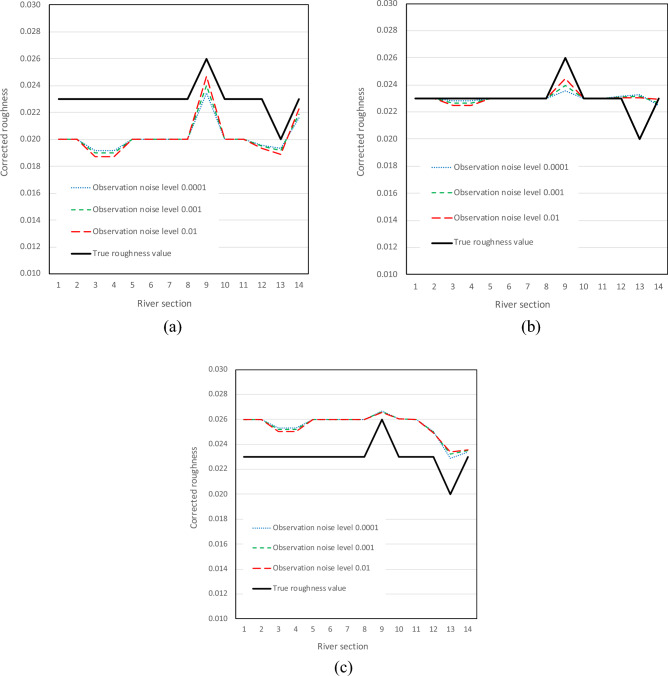
Corrected roughness values at each river section at the setting of initial roughness value of (**a**) 0.020, (**b**) 0.023, and (**c**) 0.026.

We further analyze the corrected channel-bed roughness value at each river section calculated from the data assimilation model with the inputs of different initial roughness values (i.e., 0.020, 0.023, and 0.026). Three observation noise levels of 0.0001, 0.001 and 0.01 m are analyzed. Figure 10 shows that the corrected roughness of each river section deviates far away from the true roughness when the initial roughness value is 0.020 or 0.026, while the corrected roughness values of different river sections approach the true values when the initial roughness value is 0.023, which is equal to most river sections’ true roughness of 0.023.

The comparison study shows that the selection of initial river roughness as inputs to the data assimilation model is important for realizing a bed roughness close to the true one and thus for improving the forecasting accuracy of hydraulic state variables such as water level. This result is expected as the roughness conditions on the channel bed could significantly influence the open-channel flow and the associated transport processes in rivers, streams, and estuaries. Several methods are commonly applied to estimate river bed roughness in practice, including direct measurement of roughness elements such as grain size and sediment shape, remote sensing using satellite imagery or aerial photography^28, 29^, numerical modeling using MIKE11 or Delft3D models to simulate the flow and sediment transport processes in a river^30^, and empirical models based on empirical relationships such as Manning equation using measurable parameters (e.g., river width, slope and discharge)^20, 31^. There is no universe method for different real river networks. As such, a comparison study should be conducted to determine a cost-effective and accurate roughness estimation method.

Due to the contribution of bed roughness correction during the data assimilation process, the filtering time has become shorter to 7 h, which is more practical in the field than the filter time produced by the model without bed roughness correction. This short filtering time is comparable to other studies. For example, Karri et al.^32^ showed that their data assimilation treatment coupling with the ensemble Kalman filter and hydrodynamic model had the highest forecast quality for the forecast horizon of up to 6 h, beyond which the forecasting error started to fluctuate.

Although the experimental evaluation demonstrates that a high water-level forecasting capacity of the data assimilation model can be achieved if both water level and channel-bed roughness can be corrected in a real-time manner, the data assimilation model with only water level correction does not produce a relatively large forecasting error within the normal range of observation noise level, as shown in Fig. 4. This is because our experimental evaluation is performed in a plain river network instead of a mountain river network with turbulent flows and steep slopes (from 0.4 to 5%), which could be significantly influenced by bed roughness^33^. Future studies could be conducted on a mountain river network to test the contribution of the bed roughness correction during the data assimilation process.

The uniqueness of our research lies in the real-time correction of both channel-bed roughness and water level using the extended Kalman filter, enhancing the accuracy and reliability of the river network hydrodynamic model. In contrast to existing methods that typically correct these parameters independently, our approach considers the intricate relationship between the two, optimizing them concurrently. This joint correction led to substantial improvements in filtering time (44–63% reduction) and water level forecasting accuracy (up to 80% increase) compared to methods that only address water level. Furthermore, our model demonstrates greater resilience to observation noise, presenting a lower increase in filtering time and forecasting error as noise increased. It even achieved negligible water level forecasting error after seven hours of data assimilation with accurate hydraulic state measurements. Our study is innovative in that it introduces an algorithm enabling real-time correction of roughness, thereby improving model performance significantly, particularly useful in the realm of flood forecasting.

Roughness and water level are crucial variables in the hydrodynamic modeling process. Their accurate estimation and correction significantly impact the accuracy and reliability of the model. Notably, roughness, as a model parameter, and water level, as a hydraulic state variable, often exhibit strong mutual influence, thus justifying their joint optimization. It is important to note, however, that while water temperature, climatic conditions, and other variables undoubtedly impact the hydrodynamics of a river network, incorporating all these factors simultaneously would result in an exceedingly complex model. The integration of these parameters could lead to the model becoming computationally intensive and potentially intractable. Therefore, we have chosen to focus our research on two of the most influential parameters—roughness and water level—to balance the trade-off between model complexity and computational efficiency. We believe that optimizing these two variables constitutes a meaningful and significant step towards improving hydrodynamic model accuracy and reliability. In future work, we aim to incrementally integrate more variables into the model, provided they can enhance the predictive capabilities without overly complicating the model.

It is also crucial to highlight a key limitation encountered in our methodology. While our method exhibits the capability to effectively track true roughness in various scenarios, its performance is significantly influenced by the accuracy of the initial roughness value set. Specifically, as demonstrated in Fig. 10, if the initial roughness is set to values such as 0.020 or 0.026, the updating process may not converge to the true roughness value of 0.023. Consequently, the accuracy in predicting roughness is dependent on ensuring that the initial roughness value is set with precision. Potential users of this methodology should be cognizant of this aspect and consider the necessity for trial-and-error tests to ascertain the most accurate initial roughness for their specific scenarios.

In this study, the traditional extended Kalman filter coupling with the river network hydraulic model is used to carry out the data assimilation with the real-time correction of both hydraulic state variables and model parameters. Given that Kalman filter coupling with hydraulic models is one of the efficient methods to adjust real-time flood series for reducing forecasting errors^4, 5, 34^, future studies can be carried out to compare other improved types of Kalman filters, such as ensemble Kalman filter^32, 35^ and traceless Kalman filter^36^ in terms of model forecasting capacity.

## Conclusion

In this paper, we develop a new data assimilation model that uses the extended Kalman filtering method to simultaneously correct both the channel-bed roughness and water level. These are the key model parameters and state variables of a river network hydrodynamic model. The influence of the initial roughness and observation/measurement noise level on the model performance in terms of water level forecasting accuracy and filtering time is investigated thorough experimental evaluation and parameter analysis. The results are also compared to the data assimilation model with only real-time correction of state variable, i.e., water level in this study. The following conclusions can be obtained:

1. Regardless of the observation noise level, both filtering time and water level forecasting error are significantly reduced in the data assimilation model that corrects both the water level and channel-bed roughness, compared to the model that only corrects the water level. In the case of a real river network, filtering time is reduced significantly by 44–63%. The RMSE values for water level forecasting are substantially decreased by up to 80%.
2. Both filtering time and water level forecasting error increase with the observation noise level for both models, regardless of the initial roughness. However, the increase is much lower for the data assimilation model that corrects both the water level and channel-bed roughness, compared to the model that only corrects the water level. With relatively accurate measurements of hydraulic state variables in practice (e.g., with a measurement error level of 0.005 m), the model can forecast the state variables well with a negligible error after around 7 h of data assimilation.
3. It is important to note a limitation of our method: the accuracy of tracking true roughness is highly dependent on the initial roughness value set. As shown in Fig. 10, when the initial values were set to either 0.020 or 0.026, the method struggled to converge to the true roughness of 0.023. Users of this approach should be aware of this dependency and the potential need for trial-and-error in setting the initial roughness accurately. The water level forecasting error can be further reduced if the initial channel-bed roughness at each river section is closer to the true value. The impact of the initial roughness values on the model's forecasting performance is independent of the measurement errors. An optimal channel-bed roughness value is thus crucial for improving water level forecasting in practice.

## Data Availability

All data, models, and code generated or used in this study are available upon request from the corresponding author.
